# Adolescent anxiety and depression: burden of disease study in 53,894 secondary school pupils in the Netherlands

**DOI:** 10.1186/s12888-022-03868-5

**Published:** 2022-03-30

**Authors:** Leonie Klaufus, Eva Verlinden, Marcel van der Wal, Pim Cuijpers, Mai Chinapaw, Filip Smit

**Affiliations:** 1 Department of Epidemiology, Health Promotion and Health Care Innovation, Public Health Service Amsterdam, Nieuwe Achtergracht 100, 1018WT Amsterdam, the Netherlands; 2 Department of Public and Occupational Health, Amsterdam Public Health research institute, Amsterdam UMC, Vrije Universiteit, Amsterdam, the Netherlands; 3 Department of Clinical, Neuro and Developmental Psychology, Amsterdam Public Health research institute, Vrije Universiteit Amsterdam, Amsterdam, the Netherlands; 4 Department of Epidemiology and Biostatistics, Amsterdam Public Health research institute, Amsterdam UMC, Location VUmc, Amsterdam, the Netherlands; 5 Department of Mental Health and Prevention, Trimbos Institute (Netherlands Institute of Mental Health and Addition), Utrecht, the Netherlands

**Keywords:** Depressive Disorder, Anxiety disorders, Disease burden, Quality of Life, Public health, Adolescents

## Abstract

**Background:**

Prevalence rates of anxiety and depression in adolescence are rising markedly in early adolescence. It is important to quantify the non-fatal disease burden of anxiety and depression, such that early interventions can be well targeted, and resources can be allocated in a just and optimal way. This study aimed to estimate the non-fatal disease burden of anxiety and depression with and without suicidal ideation in girls and boys aged 13, 14, and 15 years.

**Methods:**

Participants were 53,894 secondary school pupils who completed health questionnaires between September 2018 and July 2019. A design-based approach was used for complex survey data with post-stratification weights and taking clustering at school-level into account. At individual level, disability weights (DWs) were calculated for each disorder. At population level, DWs were multiplied by the point-prevalence per one thousand population of the respective disorders to compute years lived with disability (YLD). DWs and YLD of anxiety and depression were calculated with and without adjustment for comorbid eating disorders, substance use disorders and somatic illnesses.

**Results:**

The unadjusted DW of depression with suicidal ideation (0.30) was greater than without suicidal ideation (0.26), and both were greater than the DW of anxiety (0.24). A similar ranking was obtained after adjusting for comorbidities. At population level, where the prevalence of the disorders come into play, the YLD disease burden was greatest for anxiety, followed by depression with suicidal ideation and depression without suicidal ideation with 17.40, 9.85, and 5.28 YLD per one thousand population, unadjusted for comorbidities. This pattern was the same after adjustment, but then the total YLD of depression with and without suicidal ideation was similar to the YLD of anxiety (12.47 and 12.46, respectively). Girls showed a significantly greater YLD burden of anxiety and depression than boys, but no differences were found between different age groups.

**Conclusions:**

From an individual clinical perspective, depression, especially when accompanied by suicidal ideation, was identified as a major health concern, especially in girls. From a public health perspective, both anxiety and depression, especially when accompanied by suicidal ideation, were identified as major drivers of disease burden, again most notably in girls.

**Supplementary Information:**

The online version contains supplementary material available at 10.1186/s12888-022-03868-5.

## Background

Prevalence rates of anxiety and depression in adolescence are rising markedly, especially in early adolescence [1–3]. At age 14, around 38% of youth in a general population have developed an anxiety disorder and 3.1% a depressive disorder at least once in their lives [3]. Worldwide prevalence rates among children and adolescents are 6.5% for any anxiety disorder and 2.6% for any depressive disorder [4]. Although anxiety disorders are more prevalent, depressive disorders cause more distress and impairment of daily activities [2, 5, 6], especially when depression is accompanied by suicidal ideation [7]. Presence of comorbid disorders and illnesses may further erode quality of life [2]. Approximately 40% of adolescents with one disorder also suffer from another disorder [8, 9], with a fourfold risk of developing both anxiety and depression, and a threefold risk of developing both depression and substance use disorders [10]. To ameliorate the health loss (also referred to as disability [11]) due to adolescent anxiety and depression and its possible comorbidities, deploying early intervention before or at first onset of these disorders has been suggested [3, 12–14].

Individuals and health systems spend enormous resources on interventions preventing, improving, or curing disability [11]. Therefore, Vos and colleagues (2012) stated that “some form of periodic accounting about the burden of non-fatal illness in populations […] should be available for policy making and planning” [11]. Hence, it is important to quantify the non-fatal disease burden associated with anxiety and depression, such that early interventions can be well targeted, and resources can be allocated in a just and optimal way [15].

Disease burden can be quantified in several ways. First, at individual level as *disability weights* (DWs). A DW is a quantification of the severity of health loss associated with a disease on a scale from 0 (*no disability*) to 1 (*completely disabled*). DWs are important for clinicians as they portray a realistic picture of how much impairment one suffers from an adverse health state in daily life. Second, at population level as *years lived with disability* (YLD) per one thousand population. YLD are important for public health professionals as these metrics incorporate the number of people affected by a disease multiplied by the DW of that disease. When adjusting for comorbidities, one gets an idea of the unique contribution of a disease to the total burden of all comorbid diseases in a population.

Few studies have quantified the burden of diseases in children and youth. Gore and colleagues [16] and Erskine and colleagues [1] found that at population level, mental and substance use disorders were the leading causes of disease burden in children and youth worldwide. Major depressive disorder caused the highest disease burden in both girls and boys, whereas anxiety disorders caused the second highest disease burden in girls and the third highest in boys when adjusting for comorbidities, with disease burden being significantly greater in girls than in boys [1]. However, as policy making and planning occurs per country, it is important to assess the disease burden nationally. In addition, in Erskine and colleagues’ study [1], disability weights for adolescent disorders were obtained from adults. Assessing disability directly from a large sample of adolescents is important, thereby acknowledging adolescents as the rightful judges of their own mental health [17]. In addition, Erskine and colleagues [1] did not quantify the burden of diseases unadjusted for comorbidities, which gives a descriptive picture of how much one suffers from a disorder in daily life, nor did they differentiate between different ages or between major depressive disorder with and without suicidal ideation when examining the burden of disease, while previous research has shown that the traits of depression with suicidal ideation are (phenotypically) different from depression without suicidal ideation [7].

This study aims to assess the disease burden of adolescent anxiety and depression with and without suicidal ideation at individual and population level, with and without adjusting for comorbidities, and disaggregated by gender and age in the Netherlands. Following Salomon and colleagues [6], we hypothesize that at individual level, the DW of depression is greater than the DW of anxiety (H1). In addition, we hypothesize that the DW of depression with suicidal ideation is greater than of depression without suicidal ideation (H2). Next, based on the study of Erskine and colleagues [1], we hypothesize that YLD of depression is greater than of anxiety (H3). Further, following Erskine and colleagues [1] and Salk and colleagues [18], we expect girls to show a greater YLD disease burden of anxiety and depression than boys (H4). Finally, we hypothesize that older adolescents show a greater YLD disease burden of anxiety and depression than younger adolescents (H5) (cf. Solmi and colleagues [3]).

## Methods

### Participants

Participants were 53,894 adolescents in the age range of 13 to 15 years. These adolescents participated in a public health screening, conducted by professionals from seven Youth Health Care services in the Netherlands. These Youth Health Care services are located in seven different urban and rural regions. In these regions, almost all secondary schools for practical education, lower vocational education, higher general secondary education, and pre-university education (327 schools in total) participated in the screening between September 2018 and July 2019, with the exception of a few schools for religious reasons and a few schools for practical education. Prior to screening, pupils and their parents received extensive information about the screening, and a total of 53,941 pupils provided written informed consent and participated in the ensuing assessment. Of these, 53,894 participants were included in the current study because they had complete data on gender, age, and ethnicity. Under Dutch law, no approval is needed from a medical ethics review committee for re-use of anonymous data obtained in health care for research purposes.

### Measures

#### Public health screening

The screening consisted of several self-report online questionnaires on mental health, substance use, physical, and social problems. Screen-positive adolescents were invited for a personal health check by a school nurse or physician and referred to a preventive intervention or treatment when required. Six of these self-report questionnaires were used in the current study and are described below.

#### KIDSCREEN-10

The KIDSCREEN-10 [19, 20] is a self-report questionnaire for children and adolescents aged 8 to 18 years old. It assesses health-related quality of life (HRQoL) in the previous week, “covering physical, emotional, mental, social, and behavioral components of wellbeing and functioning as perceived by patients and/or other individuals” [21]. The KIDSCREEN-10 consists of ten items: fit and well (KS_1), energy (KS_2), sad (KS_3), lonely (KS_4), had enough time for yourself (KS_5), been able to do things that you want to do in your free time (KS_6), parent(s) treated you fairly (KS_7), had fun with friends (KS_8), got on well at school (KS_9), and been able to pay attention (KS_10). Items are scored on a 5-point Likert scale, ranging from 1 (*never*) to 5 (*always*) and summed to a total score, ranging from 10 to 50, with higher scores indicating better HRQoL. Previous research has shown sufficient reliability (Cronbach’s α = 0.82; *ICC* = 0.70) and criterion validity (*r* = 0.91 compared to the general factor of the KIDSCREEN-52) in a large general sample (*N* = 22,830) aged 8 to 18 [19].

#### Child Health Utility 9D (CHU9D)

The CHU9D [22–24] is a self-report questionnaire for children and adolescents aged 7 to 17 assessing current HRQoL. In contrast to the KIDSCREEN-10, which provides a simple summary score, the CHU9D is a preference-based measure in which health states contain preference weights that are determined relative to each other, which has the advantage that it can be used in cost-effectiveness analyses or burden of disease studies. The CHU9D consists of nine dimensions: being worried, sad, in pain, tired, annoyed, schoolwork/homework, sleep, daily routine, and ability to join in activities. Each dimension consists of five descriptions of increasing severity levels. The CHU9D descriptions can, thus, define 5^9^ = 1,953,125 health states. Each health state is valued relative to other health states, based on the standard gamble method in an adult population [25] or on profile case best–worst scaling discrete-choice experiments in a community sample of adolescents [17]. These health state valuations are used to generate a utility value ranging from 0 (*death*) to 1 (*perfect health*). Previous studies have shown moderate to sufficient reliability (Cronbach’s α = 0.77–0.80, *ICC* = 0.65) and convergent validity (*r* = 0.57–0.69, *p* < 0.01 compared to the PedsQL; *ICC* = 0.74 compared to the HUI2) of the CHU9D utility values in general populations of adolescents (*N* = 228–1,912) [26–28].

In the current study, the CHU9D was not used, but CHU9D utilities were generated by mapping the KIDSCREEN-10 scores onto the CHU9D utilities. Mapping was necessary as the KIDSCREEN lacks preference weights for health states and is, therefore, not suitable for a burden of disease study. To map the KIDSCREEN-10 score onto the CHU9D utilities, we used Chen and colleagues’ preferred algorithm that showed the best goodness-of-fit results (mean absolute error = 0.0937; root mean square error = 0.1193) [29].

#### Revised Child Anxiety and Depression Scale short version (RCADS-22)

The RCADS-22 [30–32] is a questionnaire for children and adolescents aged 8 to 18. It assesses “broad” anxiety (i.e., separation anxiety disorder, social phobia, generalized anxiety disorder, panic disorder, and obsessive-compulsive disorder) with 15 items and depression with seven items that are based on DSM-IV criteria. Items are scored according to the frequency of their occurrence on a 4-point Likert scale, ranging from 0 (*never*) to 3 (*always*) and summed to total scores. The total anxiety score ranges from 0 to 45, and the total depression score ranges from 0 to 21. Higher scores are indicative of higher levels of anxiety and depression. A previous study by Klaufus and colleagues [32] found that a raw anxiety score of 14 and above can be used as the clinical cut-off for an anxiety disorder with sensitivity = 75% and specificity = 76% in a Dutch general school sample; however, Klaufus and colleagues were unable to indicate a cut-off score for depression. Therefore, we used a *T*-score of 70 and above as indicative of having a clinically relevant depressive disorder, which was revealed in another study in a U.S. sample [31], corresponding to a raw depression cut-off score of 10 in a representative Dutch sample [33].

#### Ask Suicide-screening Questions (ASQ)

The ASQ [34] is a short questionnaire assessing risk for suicide in youth and young adults. It consists of four items: three items measure current suicidal ideation and one item measures a previous suicide attempt. Items are scored 0 (*no*) or 1 (*yes*). A positive response to at least one question is usually considered as “at risk” for suicide [34]. In this study, we used the three items measuring current suicidal ideation and considered a positive response to at least one question as having suicidal ideation. In a general sample of school adolescents (*N* = 84, aged 12 to 17), the sensitivity and specificity of this 3-item ASQ were 84% and 82%, respectively, compared to two suicidal ideation items in the semi-structured diagnostic interview Schedule for Affective Disorders and Schizophrenia for School-Age Children Present and Lifetime Version [35, 36] (results not published).

#### SCOFF (eating disorder) questionnaire

The SCOFF [37] is a short self-report questionnaire for adolescents and adults assessing the potential existence of an eating disorder with five items. These five items form the acronym “SCOFF” (“Do you make yourself **S**ick because you feel uncomfortably full?”, “Do you worry you have a lost **C**ontrol over how much you eat?”, “Have you recently lost more than **O**ne stone in a 3 month period?”, “Do you believe yourself to be **F**at when others say you are too thin?”, “Would you say that **F**ood dominates your life?”[37]). Items are scored 0 (*no*) or 1 (*yes*) and summed to a total scale, ranging from 0 to 5. Higher scores indicate more eating problems. A previous study has indicated that a cut-off score of 3 and higher maximizes both sensitivity (i.e., 99.1%) and specificity (i.e., 95.8%) in an adult population aged 18 to 40 [38].

#### Severity of Dependence Scale (SDS)

The SDS [39] is a short self-report questionnaire for adolescents and adults assessing the degree of psychological dependence of substance use (in this study: alcohol, tobacco, and cannabis) in the previous three months. It consists of five items scored on a 4-point Likert-scale: 0 (*never*) to 3 (*always / nearly always*) for items one to four, and 0 (*not difficult*) to 3 (*impossible*) for item five. Items are summed to a total score ranging from 0 to 15; higher scores indicate higher levels of dependence. Previous studies have suggested an SDS score of 3 and above as indicative of dependence on alcohol in a general and clinical sample of youth and adults [40]. A score of 4 and above is indicative of dependence on cannabis in a general sample of adolescents [41, 42]. To our knowledge, no studies have been published in which a cut-off score is recommended for tobacco dependence. We determined to use the same cut-off score for tobacco as for cannabis dependence (i.e., 4), as smoking tobacco and using cannabis are highly correlated [43, 44].

#### Chronic Conditions Short Questionnaire (CCSQ)

The CCSQ [45, 46] is a short self-report questionnaire for youth aged 10 to 18 measuring the burden of a chronic physical illness. It consists of three items: one item assesses whether or not someone has a chronic illness diagnosed by a physician, and two items assess the consequences of the illness; that is, taking medication and/or affecting attendance and participation at school. Items are scored 0 (*no*) or 1 (*yes*). Based on the item scores, youth can be classified into three categories: 1) healthy youth; 2) youth with a chronic condition without related consequences; 3) youth with a chronic condition and related consequences [46]. A previous study has demonstrated sufficient construct validity of the CCSQ based on significant differences in the mean indexes of four validation scales (i.e., subjective complaints checklist and three subscales of the Child Health and Illness Profile—Adolescent Edition [CHIP-AE] questionnaire) between healthy and chronically-ill students with related consequences according to the CCSQ [46].

In this study, we classified youth into two categories: 1) healthy youth and youth with a chronic condition without related consequences; 2) youth with a chronic condition and related consequences, as a previous study has shown that students with a chronic disease but without related consequences do not significantly differ from students without a chronic condition in limitations of activity, physical and emotional discomfort, measured by the CHIP-AE [46].

### Analyses

All analyses were performed in Stata version 15 taking into account that sample data needed to be weighted to follow the same distribution as seen in the general population and had a “nested” structure of pupils in schools, and that missing observations in some variables needed to be imputed.

Post-stratification weights were calculated for gender, age (i.e., 13, 14, and 15 years old), and ethnicity (i.e., Dutch, western, and non-western migration background) in accordance with the Dutch guideline for weighting in public health settings [47]. The population distribution over gender, age, and ethnicity of pupils in the 2018–2019 academic year were retrieved from Statistics Netherlands [48].

In total, 1,336 respondents had missing values on one or more variables, and these were imputed using multiple (tenfold) imputation with the expectation maximization (EM) algorithm. For imputation, the following variables were used: 1) the demographic variables sex, age, and ethnicity; 2) the CHU9D utility value (see calculation below); 3) the RCADS-22 anxiety and depression scales, and the ASQ scale; and 4) the SCOFF, SDS, and CCSQ scales. A sensitivity analysis was performed using multiple (tenfold) imputation with chained equations (MICE) instead of imputation based on EM for cross-validation.

Utility scores were estimated by mapping the KIDSCREEN-10 index onto the CHU9D utility scores using Chen and colleagues’ preferred mapping algorithm [29]. The preferred algorithm was an *MM*-estimator with stepwise-selected KIDSCREEN-10 item scores, which had the best predictive accuracy of CHU9D utility, based on profile case best–worst scaling discrete-choice experiments in a community-based adolescent sample [17]: CHU9D utility score = 0.222655 + 0.037867 * KS_1 + 0.023085 * KS_2 + 0.037192 * KS_3 + 0.021284 * KS_4 + 0.024877 * KS_9 + 0.022256 * KS_10 [29]. Utility values > 1 were truncated at 1 in line with the recommendations of Chen and colleagues [29], leading to a range in utility scores from 0.39 to 1.

To examine our hypotheses, the multiply imputed data were analyzed using a design-based approach for complex survey data with the post-stratification weights and taking clustering of pupils in schools into account. All analyses were performed for girls and boys, and for the ages 13, 14 and 15, separately and combined.

Assessing disease burden of anxiety and depression was done at individual and population level. At individual level, HRQoL losses were measured by disability weights (DWs), where DWs were computed as the complement of utility, U, i.e., DW = 1 – U, and the mean DW describes the level of disability as a result of a disorder ranging from 0 (*no disability*) to 1 (*completely disabled*). At population level, the disease burden was expressed as years lived with disability (YLD). YLD were calculated by multiplying the DW of a disorder by the point prevalence of that disorder to capture the number of person-years spent in illness per one thousand population [1, 11].

Finally, all analyses were based on raw (unadjusted) DWs as well as on DWs that were adjusted for comorbidities. Adjusted DWs were calculated by linear regression analyses, performed in two phases. The DW of one disorder was adjusted for presence of all other comorbid disorders in the sample, plus somatic illnesses that were only available in a subsample of *N* = 33,178.

We considered non-overlapping 95% confidence intervals as a significant difference and a difference in DWs ≥ 0.04 as an important clinical difference [49]. An important clinical difference can be defined as the smallest difference in score in the construct of interest (in this case HRQoL) which patients perceive as beneficial [49].

## Results

### Sample characteristics

Table 1 shows the characteristics of the two study samples compared to the distribution in the Dutch population. Deviations from the population data ranged from 0.10% to 4.77% in the total sample and from 0.51% to 7.54% in the subsample. After weighting, the distribution over age, sex, and ethnicity of the samples were the same as in the Dutch population.

**Table 1:** Demographics of the 13–15 year olds in the Dutch population, total sample, and subsample^a^

	**Dutch population** **(*N* = 565,921)**	**Total sample** **(*N* = 53,894)**	**Subsample** **(*N* = 33,178)**
Gender			
Girls (%)	49.7	50.5	50.2
Age			
13 (%)	32.1	29.1	26.5
14 (%)	33.5	36.6	41.0
15 (%)	34.4	34.3	32.6
Ethnicity			
Dutch (%)	75.3	71.0	78.6
Western^b^ migration background (%)	7.12	6.7	5.3
Non-western^c^ migration background (%)	17.6	22.4	16.1

^a^ The subsample completed (next to health-related quality of life, anxiety, depression, suicidal ideation, eating disorders, and substance use disorders) a questionnaire on somatic illnesses

^b^ Western = Europe (excluding Turkey), North America, Oceania, Japan, Indonesia

^c^ Non-western = Africa, Latin-America, Asia (without Japan and Indonesia)

### Disability weights unadjusted for comorbidities

Table 2 presents the DWs of anxiety and depression unadjusted for comorbidities (see Additional file 1 for the unadjusted characteristics of disease burden of anxiety and depression disaggregated by gender and age). In the total sample, the unadjusted DW of anxiety was 0.24 (95% CI = 0.24–0.24). The DW of depression without suicidal ideation was significantly greater (0.26, 95% CI = 0.25–0.26). Moreover, the DW of depression with suicidal ideation (0.30, 95% CI = 0.29–0.30) was significantly greater than the DW of depression without suicidal ideation.

**Table 2:** Unadjusted characteristics of disease burden of anxiety and depression (*N* = 53,894)

Gender	Disorder	DW (95% CI)	Pyrs/1000 (95% CI)	YLD/1000 (95% CI)
Girls	Anxiety	0.25 (0.24–0.25)	112.27 (106.38–118.16)	28.07 (22.07–34.07)
	Depression with suicidal ideation	0.31 (0.30–0.31)	50.47 (46.79–54.14)	15.65 (11.94–19.35)
	Depression without suicidal ideation	0.27 (0.27–0.28)	28.53 (26.27–30.79)	7.70 (5.43–9.98)
Boys	Anxiety	0.21 (0.20–0.22)	33.26 (30.58–35.94)	6.98 (4.29–9.68)
	Depression with suicidal ideation	0.27 (0.26–0.28)	15.39 (13.54–17.24)	4.16 (2.30–6.01)
	Depression without suicidal ideation	0.23 (0.22–0.24)	12.20 (10.72–13.67)	2.81 (1.33–4.28)
All	Anxiety	0.24 (0.24–0.24)	72.52 (68.71–76.33)	17.40 (13.56–21.25)
	Depression with suicidal ideation	0.30 (0.29–0.30)	32.82 (30.48–35.16)	9.85 (7.50–12.19)
	Depression without suicidal ideation	0.26 (0.25–0.26)	20.31 (18.90–21.73)	5.28 (3.86–6.70)

***Note:*** *DW* Disability Weights, *Pyrs/1000* Person Years per one thousand population, *YLD/1000* Years Lived with Disability per one thousand population, *CI* Confidence Interval

### Disability weights adjusted for comorbidities

Table 3 presents the DWs of anxiety, depression with and without suicidal ideation, eating disorders, dependence of alcohol, tobacco, and cannabis, and physical illnesses, adjusted for comorbidities (see Additional file 2 for the adjusted characteristics of disease burden of mental disorders and physical illnesses disaggregated by gender and age). Although the adjusted DWs of anxiety and depression with and without suicidal ideation were smaller than the unadjusted DWs, the results of the hypotheses examinations remained similar. In other words, the DW of depression without suicidal ideation (0.22, 95% CI = 0.21–0.23) was significantly greater than the DWs of anxiety in the total sample and subsample (0.18 and 0.17, respectively, 95% CIs = 0.17–0.18). In addition, the DW of depression with suicidal ideation (0.24, 95% CI = 0.24–0.25) remained significantly greater than the DW of depression without suicidal ideation in the total sample and subsample.

**Table 3:** Adjusted characteristics of disease burden of mental disorders and physical illnesses

**Gender**	**Disease**	**Total sample (*N* = 53,894)**			**Subsample (*N* = 33,178)**		
		**DW** **(95% CI)**	**Pyrs/1000** **(95% CI/1000)**	**YLD/1000** **(95% CI/1000)**	**DW** **(95% CI)**	**Pyrs/1000** **(95% CI/1000)**^**a**^	**YLD/1000** **(95% CI/1000)**
Girls	Anxiety	0.19 (0.18–0.19)	112.27 (106.38–118.16)	20.88 (14.88–26.89)	0.18 (0.17–0.19)		20.26 (14.17–26.36)
	Depression with suicidal ideation	0.26 (0.25–0.26)	50.47 (46.79–54.14)	12.93 (9.22–16.64)	0.25 (0.25–0.26)		12.80 (9.06–16.54)
	Depression without suicidal ideation	0.23 (0.23–0.24)	28.53 (26.27–30.79)	6.68 (4.40–8.96)	0.23 (0.23–0.24)		6.66 (4.37–8.95)
	Eating disorder	0.16 (0.16–0.17)	73.54 (69.01–78.07)	12.02 (7.44–16.61)	0.16 (0.15–0.16)		11.59 (6.95–16.23)
	Dependence of alcohol	0.15 (0.13–0.16)	3.89 (3.08–4.70)	0.58 (-0.22–1.39)	0.15 (0.13–0.17)		0.57 (-0.23–1.38)
	Dependence of tobacco	0.18 (0.17–0.19)	10.61 (8.80–12.42)	1.91 (0.10–3.71)	0.18 (0.16–0.19)		1.87 (0.06–3.68)
	Dependence of cannabis	0.15 (0.11–0.19)	0.73 (0.39–1.06)	0.11 (-0.22–0.45)	0.17 (0.11–0.23)		0.12 (-0.22–0.46)
	Physical illnesses				0.14 (0.13–0.14)	115.70 (110.11–121.28)	15.62 (9.81–21.43)
Boys	Anxiety	0.16 (0.16–0.17)	33.26 (30.58–35.94)	5.48 (2.78–8.17)	0.16 (0.15–0.16)		5.24 (2.53–7.95)
	Depression with suicidal ideation	0.23 (0.22–0.24)	15.39 (13.54–17.24)	3.56 (1.71–5.41)	0.23 (0.22–0.24)		3.53 (1.68–5.39)
	Depression without suicidal ideation	0.20 (0.19–0.21)	12.20 (10.72–13.67)	2.45 (0.98–3.93)	0.20 (0.19–0.22)		2.50 (1.02–3.98)
	Eating disorder	0.13 (0.12–0.13)	19.94 (17.79–22.09)	2.52 (0.37–4.67)	0.13 (0.12–0.14)		2.66 (0.50–4.81)
	Dependence of alcohol	0.09 (0.08–0.11)	4.68 (3.56–5.80)	0.44 (-0.68–1.56)	0.09 (0.08–0.10)		0.42 (-0.70–1.54)
	Dependence of tobacco	0.13 (0.12–0.14)	9.03 (7.32–10.74)	1.15 (-0.56–2.86)	0.12 (0.11–0.13)		1.08 (-0.63–2.79)
	Dependence of cannabis	0.11 (0.08–0.14)	1.84 (1.31–2.36)	0.21 (-0.32–0.74)	0.13 (0.10–0.16)		0.24 (-0.29–0.77)
	Physical illnesses				0.10 (0.10–0.11)	91.20 (86.13–96.28)	9.23 (4.00–14.45)
All	Anxiety	0.18 (0.17–0.18)	72.52 (68.71–76.33)	12.93 (9.08–16.78)	0.17 (0.17–0.18)		12.46 (8.58–16.34)
	Depression with suicidal ideation	0.24 (0.24–0.25)	32.82 (30.48–35.16)	8.04 (5.69–10.39)	0.24 (0.24–0.25)		7.98 (5.63–10.34)
	Depression without suicidal ideation	0.22 (0.21–0.23)	20.31 (18.90–21.73)	4.47 (3.05–5.89)	0.22 (0.21–0.23)		4.49 (3.06–5.92)
	Eating disorders	0.15 (0.15–0.16)	46.58 (43.78–49.37)	7.10 (4.29–9.91)	0.15 (0.14–0.15)		6.98 (4.15–9.80)
	Dependence of alcohol	0.12 (0.11–0.13)	4.29 (3.52–5.05)	0.50 (-0.26–1.27)	0.11 (0.10–0.13)		0.48 (-0.28–1.25)
	Dependence of tobacco	0.15 (0.15–0.16)	9.81 (8.38–11.25)	1.51 (0.07–2.94)	0.15 (0.14–0.16)		1.44 (0.01–2.88)
	Dependence of cannabis	0.12 (0.10–0.14)	1.28 (0.94–1.63)	0.15 (-0.20–0.51)	0.13 (0.10–0.16)		0.17 (-0.19–0.52)
	Physical illnesses				0.12 (0.11–0.12)	103.37 (99.53–107.21)	12.26 (8.28–16.23)

***Note:*** *DW* Disability Weights, *Pyrs/1000* Person Years per one thousand population, *YLD/1000* Years Lived with Disability per one thousand population, *CI* Confidence Interval

^a^ For the calculation of years lived with disability in the subsample, the person years per one thousand population of the total sample were used for each disorder, with the exception of physical illnesses, which was only completed in the subsample

### Years lived with disability unadjusted for comorbidities

Table 2 presents the point prevalence of anxiety and depression with and without suicidal ideation expressed in number of person-years spent in illness per one thousand population, and the YLD per one thousand population, unadjusted for comorbidities (see Additional file 1 for the unadjusted characteristics of disease burden of anxiety and depression disaggregated by gender and age). It appeared that anxiety disorders showed the greatest YLD (17.40, 95% CI = 13.56–21.25 per one thousand), followed by depression with suicidal ideation (9.85, 95% CI = 7.50–12.19 per one thousand) and depression without suicidal ideation (5.28, 95% CI = 3.86–6.70 per one thousand). Girls showed significantly greater YLD due to anxiety (28.07, 95% CI = 22.07–34.07 per one thousand) and depression with and without suicidal ideation (15.65, 95% CI = 11.94–19.35; 7.70, 95% CI = 5.43–9,98 per one thousand, respectively) than boys (6.98; 95% CI = 4.29–9.68; 4.16, 95% CI = 2.30–6.01; and 2.81, 95% CI = 1.33–4.28 per one thousand, respectively). Furthermore, YLD of anxiety and depression with and without suicidal ideation did not differ significantly between the ages for both girls and boys.

### Years lived with disability adjusted for comorbidities

Table 3 shows the point prevalence of diseases expressed in number of person-years spent in illness per one thousand population, and the YLD per one thousand population for anxiety, depression with and without suicidal ideation, eating disorders, dependences on alcohol, tobacco, and cannabis, and physical illnesses, adjusted for comorbidities (see Additional file 2 for the adjusted characteristics of disease burden of mental disorders and physical illnesses disaggregated by gender and age). Adjusted YLD were smaller than unadjusted YLD, but again, the results of the hypotheses examinations remained similar. Anxiety showed the greatest YLD (12.93, 95% CI = 9.08–16.78 per one thousand), followed by depression with suicidal ideation (8.04, 95% CI = 5.69–10.39 per one thousand) and depression without suicidal ideation (4.47, 95% CI = 3.05–5.89 per one thousand) when adjusting for eating disorders and substance dependences in the total sample. When adding physical illnesses to the regression model, a similar ranking in YLD was obtained (anxiety: 12.46, 95% CI = 8.58–16.34; depression with suicidal ideation: 7.98, 95% CI = 5.63–10.34; depression without suicidal ideation: 4.49, 95% CI = 3.06–5.92 per one thousand). Interestingly, when adjusting for comorbid mental disorders, substance use disorders, and somatic illnesses, the total YLD of depression (with *and* without suicidal ideation) was similar to the YLD of anxiety (12.46 for anxiety and 12.47 for depression per one thousand).

Girls had significantly higher YLD that can be attributed to anxiety and to depression with and without suicidal ideation in both the total sample (20.88, 95% CI = 14.88 – 26.89; 12.93, 95% CI = 9.22–16.64; and 6.68, 95% CI = 4.40–8.96 per one thousand, respectively) and subsample (20.26, 95% CI = 14.17–26.36; 12.80, 95% CI = 9.06–16.54; and 6.66, 95% CI = 4.37–8.95 per one thousand, respectively) than boys (5.48, 95% CI = 2.78–8.17; 3.56, 95% CI = 1.71–5.41; and 2.54, 95% CI = 0.98–3.93 per one thousand, respectively, in the total sample; 5.24, 95% CI = 2.53–7.95; 3.53, 95% CI = 1.68–5.39; and 2.50, 95% CI = 1.02–3.98 per one thousand, respectively in the subsample). Furthermore, YLDs of both anxiety and depression with and without suicidal ideation did not differ significantly between the ages for both girls and boys.

## Discussion

### Key findings

This study estimated the disability weights (DWs) and years lived with disability (YLD) of anxiety and depression with and without suicidal ideation in 53,894 secondary school pupils aged 13 to 15 in the Netherlands. DWs and YLD were assessed for each disorder separately as well as adjusted for comorbidities.

At individual level, we hypothesized that the unadjusted and adjusted DWs of depression are greater than the DWs of anxiety (H1). In addition, we hypothesized that the unadjusted and adjusted DW of depression with suicidal ideation is greater than of depression without suicidal ideation (H2). Both hypotheses were supported by the data.

At population level, we hypothesized that YLD per one thousand population of depression is greater than of anxiety (H3). This hypothesis was not supported by the data. The unadjusted YLD of anxiety was greater than of depression (with and without suicidal ideation), and when adjusting for comorbidities, the YLD of anxiety was similar to the total YLD of depression with and without suicidal ideation. In addition, we hypothesized that girls show a greater YLD disease burden of anxiety and depression than boys (H4). This hypothesis was supported by the data. Finally, we hypothesized that older adolescents show a greater YLD disease burden of anxiety and depression than younger adolescents (H5). This hypothesis was rejected as YLD did not differ significantly across the ages.

### Findings in context

At individual level, depression showed a significantly greater disease burden than anxiety, which was consistent with the findings of Salomon and colleagues [6] and Lokkerbol and colleagues [5] in adult samples. However, when looking at *clinically important differences*, only depression with suicidal ideation showed a greater disease burden than anxiety without adjusting for comorbidities (difference between DWs = 0.04). When adjusting for comorbidities, both depression with and without suicidal ideation showed a clinically important greater disease burden than anxiety [49]. The latter finding is explained by the fact that when adjusting for comorbidities, the decrease in DW of anxiety was larger than the decrease in DW of depression with and without suicidal ideation. This means that a bigger part of the unadjusted DW of anxiety is due to comorbid conditions.

At population level, anxiety provided the greatest attribution to disease burden without adjusting for comorbidities, driven by the higher prevalence. However, when adjusting for comorbidities, disease burden of anxiety and depression were similar in terms of YLD (H3). These outcomes are at odds with Erskine and colleagues (2015), who found that major depressive disorder showed significantly greater YLD in children and youth than anxiety disorders. A possible explanation is that differences occur in prevalence rates, given that Erskine and colleagues [1] examined YLD in children and youth worldwide, while in this study, YLD were estimated in the Netherlands where prevalence rates might be different. Another explanation is that in the study of Erskine and colleagues [1], DWs were determined by adults, while in our study, DWs were determined by adolescents. Previous research found important differences between adults and adolescents in their assessment of disability due to mental problems [17].

In contrast to our expectations, we did not find that older adolescents show a significantly greater YLD due to anxiety and depression than younger adolescents (H5). Interestingly, almost all DWs of both anxiety and depression with and without suicidal ideation did increase with age, although not always significantly (nor clinically important). In addition, only the prevalence rates of girls with depression without suicidal ideation increased significantly with age; however, this did not result in significantly increasing YLD due to wide confidence intervals. Other prevalence rates decreased with age in boys or fluctuated across the ages in both girls and boys.

There are several possible explanations for the fluctuating prevalence rates from 13 to 15 years old. With regard to anxiety, it should be noted that the anxiety measure used in this study is a composite measure, consisting of items on separation anxiety disorder, social phobia, generalized anxiety disorder, panic disorder, and obsessive-compulsive disorder (formerly considered as an anxiety disorder in the DSM-IV). These anxiety disorders differ considerably in the peak age of onset, ranging from 5.5 years old for separation anxiety disorder to 15.5 years old for panic disorder and generalized anxiety disorder [3]. Given that, for example, a separation anxiety disorder usually resolves before adolescence [50], and the finding that the nature of anxiety disorders changes across ages but not the overall rate [51], this might explain the fluctuating prevalence rates of anxiety in girls. In addition, it would probably have been more advisable to measure disease burden over a wider age range in order to be able to show an overall increase (or decrease) over the ages.

### Strengths and limitations

This study has several strengths. First, we used a large sample (*N* = 53,894) to estimate the disease burden of anxiety and depression. The large sample enabled us to examine disease burden for girls and boys and for the ages 13, 14, and 15 years old separately. Second, adolescents quantified disease burden themselves rather than adults. By taking this approach, we acknowledge young people as the rightful judges of their own mental health. Third, we were (to the best of our knowledge) the first to examine disease burden of depression with and without suicidal ideation. This is important as previous research has convincingly shown that depression with suicidal ideation is phenotypically different from depression without suicidal ideation in terms of latent variable structures to such an extent that depression with suicidality is perhaps better described as “suicidal depression”[7]. Fourth, we were able to quantify disease burden for several diseases separately as well as adjusted for comorbidities, which helps to quantify disease burden descriptively (“as is”) in a disorder and also in an inferential (i.e., causal) way (the disease burden that can be attributed to specifically that disorder). And fifth, we were able to quantify disease burden at individual as well as at population level.

This study has several limitations. First, we focused on non-fatal disease burden (i.e., morbidity) thus ignoring years of life lost (YYL) due to premature death (i.e., mortality) as we did not have this information in our dataset. Second, we used a mapping algorithm to estimate losses in HRQoL, which may have introduced some bias in the DWs of our study [29]. Third, as we used cross-sectional data, when computing YLD we used the point prevalence instead of the incidence multiplied by the time spent in a health condition. Nonetheless, this approach is well-accepted and was also used in WHO Global Burden of Disease studies [6, 52]. Fourth, we defined disorders using validated cut-offs for self-report questionnaires rather than diagnostic assessments. The use of cut-offs for self-report questionnaires might have biased the results, as most studies present a single cut-off or a subset of cut-offs that performs well in their study, but in a different study with a different sample, the best cut-off score might be different [53]. Fifth, we were unable to differentiate between the distinct anxiety disorders, as the RCADS-22 only assesses “broad” anxiety [31, 32]. Lokkerbol and colleagues [5] found that different anxiety disorders differed significantly in disease burden. Sixth, the RCADS-22 measures anxiety disorders according to the DSM-IV rather than the current DSM-5 criteria. Consequently, obsessive-compulsive disorder items were part of the “broad” anxiety scale. Seventh, the CCSQ measured the consequences of a chronic physical condition for school attendance only but not the consequences for social life and family life. Therefore, disease burden driven by a somatic illness might be underestimated. And finally, we could not adjust disease burden for other disorders in adolescents such as autism spectrum disorders.

### Implications

Even in resource rich countries we do not live in an ideal world with unrestricted financial resources and abundant capacity in the form of health care professionals for the prevention and treatment of each and every health problem. Often, choices must be made to use sparse resources in an optimal way. Quantifying disease burden is important if we wish to optimally target health care interventions to those population segments most in need, and to allocate resources accordingly. We estimated disease burden of anxiety and depression with and without suicidal ideation separately as well as adjusted for comorbidities at individual and population level. These distinctions have led to slightly different disease burden estimates, and it is important to note that these distinctions serve different purposes.

From a clinical perspective, estimates at individual level are important. According to our findings (DWs in Table 3), depression is the main driver of the total non-fatal disease burden in adolescents compared to anxiety, eating disorders, substance use disorders, and somatic illnesses. Based on these findings, clinicians are recommended to prioritize the treatment of adolescents, in particular girls, suffering from depression and especially when depression is accompanied by suicidal ideation.

That said, this prioritization of diseases at individual level might be modified for several reasons. For example, when an adolescent is admitted with multiple comorbid disorders and/or illnesses, disease burden at individual level increases (that is, the sum of the DWs of the separate comorbid disorders and illnesses in Table 3). Therefore, the disease burden of an adolescent with multiple comorbid disorders might be greater than the disease burden of an adolescent with depression only, and in that case, clinicians are recommended to prioritize the treatment of the adolescent with multiple comorbid disorders. In addition, this study did not include excess mortality in the quantification of disease burden. Based on a former study [1], it might be expected that mortality rates are higher in eating disorders or substance use disorders than in depression. By implication, longer term prognosis–especially about the risk of premature death– needs to be factored in the judgement when it comes to the prioritization of one health condition over another for treatment.

From a public health perspective, prioritization of diseases for prevention or treatment purposes might be necessary too, as public health policy is aimed at achieving the greatest health impact in the population under the constraint of limited resources. The YLD, adjusted for comorbidities, help to identify those health conditions that are the main drivers of the total non-fatal disease burden in a population (Table 3). Based on our findings, public health professionals are recommended to prioritize the prevention or treatment of adolescents, particularly girls, with anxiety or depression, especially when depression is accompanied by suicidal ideation, over adolescents with eating disorders or substance use disorders, or, to a smaller extent, somatic illnesses. Given the result that at individual level, the disease burden of anxiety is rather low, while at population level, anxiety is one of the main drivers of disease burden, public health professionals might consider to offer effective and economically affordable interventions to adolescents with anxiety or at imminent risk of developing an anxiety disorder, for example by offering self-help interventions over the internet, as such interventions are easily disseminated in the population [5].

However, the prioritization of diseases at population level might be modified for several reasons as well. As mentioned before, this study did not adjust for mortality rates associated with the distinct disorders and illnesses. A former study has indicated that the ranking of diseases does not significantly change when adjusting disease burden, next to YLD, for YLL due to premature mortality at a global population level [1]; however, further research is needed to examine whether this is the case in a general Dutch adolescent population. Second, the prioritization of diseases might be modified when including the weighs of the long-term consequences and disease sequelae. This study quantified the YLD for youth in early adolescence. Disease burden of, for example, dependence of alcohol, tobacco, or cannabis usually develops in late adolescence and increases at an accelerated pace in older ages when the adverse effects of substance use become more pronounced [3]. From a public health perspective, it might, thus, be wise to prioritize the prevention of substance use disorders based on the disease burden later in life.

## Conclusions

In Dutch adolescents, the non-fatal disease burden of depression was greater when depression was accompanied by suicidal ideation (i.e., “suicidal depression”). The disability weights of depression with and without suicidal ideation were greater than the disability weight of anxiety at individual level; however, when regarded at population level where disease burden is also driven by the prevalence, both anxiety and depression emerge as the disorders associated with the greatest non-fatal disease burden. Thus, it depends on the perspective—clinical or public health—which disorder contributed the most to the total non-fatal disease burden in secondary school pupils.

## Supplementary Information

**Additional file 1:** **Table S1. **Unadjusted characteristics of disease burden of anxiety and depression (*N *= 53,894).

**Additional file 2:** **Table S2. **Adjusted characteristics of disease burden of mental disorders and physical illnesses.

## Data Availability

The dataset used during the current study is available from the corresponding author on reasonable request.

## Abbreviations

ASQ: Ask Suicide-screening Questions
CCSQ: Chronic Conditions Short Questionnaire
CHU9D: Child Health Utility 9D
CI: Confidence interval
DSM: Diagnostic and Statistical Manual of mental disorders
DW: Disability weight
EM: Expectation maximization
H: Hypothesis
HRQoL: Health-related quality of life
ICC: Intraclass correlation coefficient
KS: KIDSCREEN
MICE: Multiple imputation with chained equations
RCADS: Revised Child Anxiety and Depression Scale
SCOFF: Eating disorder questionnaire
SDS: Severity of Dependence Scale
U: Utility
YLD: Years lived with disability
YYL: Years of life lost
