# Supplementary material for: Adolescent anxiety and depression: burden of disease study in 53,894 secondary school pupils in the Netherlands
Source: BMC Psychiatry. 2022 Mar 30;22:225. doi: 10.1186/s12888-022-03868-5 (PMC8969267; doi:10.1186/s12888-022-03868-5)
Supplement: Supplementary file 1 — Additional file 1: Table S1. Unadjusted characteristics of disease burden of anxiety and depression (N = 53,894). [file 12888_2022_3868_MOESM1_ESM.docx]

**Supplement 1**

**Adolescent anxiety and depression:**

**Burden of disease study in 53,894 secondary school pupils in the Netherlands**

**by L. Klaufus et al., 2022, *BMC Psychiatry***

**Table S1.** **Unadjusted characteristics of disease burden of anxiety and depression (*N* = 53,894)**

| Gender | Age | Disorder | DW  (95% CI) | Pyrs/1000  (95% CI/1000) | YLD/1000  (95% CI/1000) |
| --- | --- | --- | --- | --- | --- |
| Girls | 13 | Anxiety | 0.23  (0.22–0.24) | 117.89  (109.72– 126.05) | 27.11  (17.24–36.98) |
|  |  | Depression with suicidal ideation | 0.30  (0.29–0.31) | 46.57  (41.23–51.91) | 13.97  (8.57–19.37) |
|  |  | Depression without suicidal ideation | 0.26  (0.24–0.27) | 21.04  (17.80–24.29) | 5.47  (2.21–8.73) |
|  | 14 | Anxiety | 0.25  (0.24–0.26) | 120.60  (112.62–128.59) | 30.15  (21.87–38.43) |
|  |  | Depression with suicidal ideation | 0.31  (0.30–0.32) | 54.12  (48.16–60.07) | 16.78  (10.75–22.80) |
|  |  | Depression without suicidal ideation | 0.28  (0.27–0.29) | 31.61  (27.93–35.29) | 8.85  (5.14–12.56) |
|  | 15 | Anxiety | 0.26  (0.26–0.27) | 98.91  (90.59–107.23) | 25.72  (17.20–34.23) |
|  |  | Depression with suicidal ideation | 0.31  (0.30–0.32) | 50.55  (44.98–56.11) | 15.67  (10.04–21.30) |
|  |  | Depression without suicidal ideation | 0.28  (0.27–0.29) | 32.52  (28.68–36.36) | 9.11  (5.23–12.98) |
| All girls | | Anxiety | 0.25  (0.24–0.25) | 112.27  (106.38–118.16) | 28.07  (22.07–34.07) |
|  |  | Depression with suicidal ideation | 0.31  (0.30–0.31) | 50.47  (46.79–54.14) | 15.65  (11.94–19.35) |
|  |  | Depression without suicidal ideation | 0.27  (0.27–0.28) | 28.53  (26.27–30.79) | 7.70  (5.43–9.98) |
| Boys | 13 | Anxiety | 0.20  (0.19–0.21) | 40.32  (35.30–45.33) | 8.06  (2.99–13.14) |
|  |  | Depression with suicidal ideation | 0.26  (0.24–0.28) | 15.67  (12.62–18.73) | 4.07  (1.02–7.13) |
|  |  | Depression without suicidal ideation | 0.21  (0.19–0.23) | 13.09  (10.48–15.70) | 2.75  (0.13–5.37) |
|  | 14 | Anxiety | 0.21  (0.20–0.22) | 33.25  (29.51–36.98) | 6.98  (3.20–10.76) |
|  |  | Depression with suicidal ideation | 0.26  (0.24–0.29) | 15.45  (12.76–18.14) | 4.02  (1.32–6.72) |
|  |  | Depression without suicidal ideation | 0.23  (0.21–0.24) | 13.09  (10.65–15.52) | 3.01  (0.57–5.45) |
|  | 15 | Anxiety | 0.23  (0.22–0.24) | 26.67  (22.83–30.51) | 6.13  (2.28–9.99) |
|  |  | Depression with suicidal ideation | 0.28  (0.26–0.30) | 15.08  (12.11–18.05) | 4.22  (1.25–7.20) |
|  |  | Depression without suicidal ideation | 0.24  (0.22–0.26) | 10.49  (8.38–12.61) | 2.52  (0.39–4.64) |
| All boys | | Anxiety | 0.21  (0.20–0.22) | 33.26  (30.58–35.94) | 6.98  (4.29–9.68) |
|  |  | Depression with suicidal ideation | 0.27  (0.26–0.28) | 15.39  (13.54–17.24) | 4.16  (2.30–6.01) |
|  |  | Depression without suicidal ideation | 0.23  (0.22–0.24) | 12.20  (10.72–13.67) | 2.81  (1.33–4.28) |
| All |  | Anxiety | 0.24  (0.24–0.24) | 72.52  (68.71–76.33) | 17.40  (13.56–21.25) |
|  |  | Depression with suicidal ideation | 0.30  (0.29–0.30) | 32.82  (30.48–35.16) | 9.85  (7.50–12.19) |
|  |  | Depression without suicidal ideation | 0.26  (0.25–0.26) | 20.31  (18.90–21.73) | 5.28  (3.86–6.70) |

**Note.** DW = Disability weights; Pyrs/1000 = person years per thousand population; YLD/1000 = years lived with disability per thousand population; CI = confidence interval.
