# Supplementary material for: Adolescent anxiety and depression: burden of disease study in 53,894 secondary school pupils in the Netherlands
Source: BMC Psychiatry. 2022 Mar 30;22:225. doi: 10.1186/s12888-022-03868-5 (PMC8969267; doi:10.1186/s12888-022-03868-5)
Supplement: Supplementary file 2 — Additional file 2: Table S2. Adjusted characteristics of disease burden of mental disorders and physical illnesses. [file 12888_2022_3868_MOESM2_ESM.docx]

**Supplement 2**

**Adolescent anxiety and depression:**

**Burden of disease study in 53,894 secondary school pupils in the Netherlands**

**by L. Klaufus et al., 2022, *BMC Psychiatry***

**Table S2. Adjusted characteristics of disease burden of mental disorders and physical illnesses**

|  |  |  | Total sample (*N* = 53,894) | | | Subsample (*N* = 33,178) | | |
| --- | --- | --- | --- | --- | --- | --- | --- | --- |
| **Gender** | **Age** | **Disease** | **DW**  **(95% CI)** | **Pyrs/1000**  **(95% CI/1000)** | **YLD/1000**  **(95% CI/1000)** | **DW**  **(95% CI)** | **Pyrs/1000**  **(95% CI/1000)^a^** | **YLD/1000**  **(95% CI/1000)** |
| Girls | 13 | Anxiety | 0.18  (0.17–0.18) | 117.89  (109.72–126.05) | 20.92  (12.40–29.43) | 0.17  (0.16–0.18) |  | 20.31  (11.50–29.12) |
|  |  | Depression with suicidal ideation | 0.24  (0.23–0.25) | 46.57  (41.23–51.91) | 11.38  (5.97–16.78) | 0.25  (0.23–0.26) |  | 11.55  (6.07–17.02) |
|  |  | Depression without suicidal ideation | 0.21  (0.20–0.22) | 21.04  (17.80–24.29) | 4.46  (1.20–7.73) | 0.22  (0.20–0.24) |  | 4.59  (1.30–7.88) |
|  |  | Eating disorder | 0.16  (0.15–0.16) | 72.50  (65.62–79.39) | 11,25  (4.22–18.29) | 0.15  (0.14–0.16) |  | 10.69  (3.52–17.87) |
|  |  | Dependence of alcohol | 0.19  (0.15–0.22) | 0.89  (0.24–1.54) | 0.17  (-0.48–0.81) | 0.20  (0.17–0.23) |  | 0.18  (-0.47–0.83) |
|  |  | Dependence of tobacco | 0.19  (0.16–0.21) | 3.02  (1.54–4.50) | 0.56  (-0.91–2.03) | 0.19  (0.16–0.23) |  | 0.57  (-0.89–2.06) |
|  |  | Dependence of cannabis | 0.08  (-0.10–0.27) | 0.20  (-0.08–0.48) | 0.02  (-0.31–0.35) | 0.11  (0.11–0.11) |  | 0.02  (-0.25–0.30) |
|  |  | Physical illnesses |  |  |  | 0.12  (0.11–0.12) | 112.17  (103.05–121.29) | 13.00  (3.36–22.64) |
|  | 14 | Anxiety | 0.19  (0.18–0.19) | 120.60  (112.62–128.59) | 22.33  (14.04–30.62) | 0.18  (0.17–0.19) |  | 21.82  (13.38–30.26) |
|  |  | Depression with suicidal ideation | 0.26  (0.25–0.27) | 54.12  (48.16–60.07) | 14.08  (8.05–20.10) | 0.26  (0.25–0.27) |  | 13.99  (7.92–20.05) |
|  |  | Depression without suicidal ideation | 0.24  (0.23–0.25) | 31.61  (27.93–35.29) | 7.48  (3.76–11.19) | 0.23  (0.22–0.24) |  | 7.32  (3.58–11.06) |
|  |  | Eating disorder | 0.16  (0.15–0.17) | 74.71  (68.35–81.06) | 12.05  (5.55–18.54) | 0.16  (0.15–0.17) |  | 11.81  (5.25–18.38) |
|  |  | Dependence of alcohol | 0.12  (0.10–0.15) | 2.98  (1.77–4.19) | 0.37  (-0.83–1.56) | 0.12  (0.09– 0.15) |  | 0.36  (-0.84–1.55) |
|  |  | Dependence of tobacco | 0.18  (0.17–0.20) | 9.68  (7.11–12.25) | 1.78  (-0.80–4.35) | 0.17  (0.16–0.19) |  | 1.69  (-0.89–4.26) |
|  |  | Dependence of cannabis | 0.14  (0.10–0.17) | 0.65  (0.13–1.17) | 0.09  (-0.42–0.60) | 0.11  (0.07–0.15) |  | 0.07  (-0.44–0.58) |
|  |  | Physical illnesses |  |  |  | 0.14  (0.13–0.14) | 115.57  (107.67–123.46) | 15.97  (7.68–24.27) |
|  | 15 | Anxiety | 0.20  (0.19–0.21) | 98.91  (90.59–107.23) | 19.66  (11.14–28.18) | 0.19  (0.18–0.20) |  | 19.06  (10.37–27.75) |
|  |  | Depression with suicidal ideation | 0.26  (0.25–0.27) | 50.55  (44.98–56.11) | 13.17  (7.54–18.81) | 0.25  (0.24–0.26) |  | 12.73  (7.03–18.43) |
|  |  | Depression without suicidal ideation | 0.24  (0.23–0.25) | 32.52  (28.68–36.36) | 7,95  (4.07–11.82) | 0.24  (0.23–0.26) |  | 7.88  (3.97–11.80) |
|  |  | Eating disorder | 0.17  (0.17–0.18) | 73.37  (66.28–80.46) | 12.75  (5.54–19.96) | 0.17  (0.16–0.18) |  | 12.22  (4.91–19.54) |
|  |  | Dependence of alcohol | 0.16  (0.14–0.18) | 7.58  (5.70–9.45) | 1,20  (-0.67–3.07) | 0.15  (0.13–0.17) |  | 1.15  (-0.72–3.03) |
|  |  | Dependence of tobacco | 0.18  (0.16–0.19) | 18.60  (14.81–22.40) | 3.31  (-0.49–7.12) | 0.17  (0.16–0.19) |  | 3.23  (-0.58–7.05) |
|  |  | Dependence of cannabis | 0.17  (0.11–0.23) | 1.29  (0.49–2.08) | 0.22  (-0.56–1.01) | 0.21  (0.14–0.28) |  | 0.27  (-0.52–1.06) |
|  |  | Physical illnesses |  |  |  | 0.15  (0.14–0.16) | 119.12  (109.15–129.08) | 17.69  (7.30–28.08) |
| All girls | | Anxiety | 0.19  (0.18–0.19) | 112.27  (106.38–118.16) | 20.88  (14.88–26.89) | 0.18  (0.17–0.19) |  | 20.26  (14.17–26.36) |
|  |  | Depression with suicidal ideation | 0.26  (0.25–0.26) | 50.47  (46.79–54.14) | 12.93  (9.22–16.64) | 0.25  (0.25–0.26) |  | 12.80  (9.06–16.54) |
|  |  | Depression without suicidal ideation | 0.23  (0.23–0.24) | 28.53  (26.27–30.79) | 6.68  (4.40–8.96) | 0.23  (0.23–0.24) |  | 6.66  (4.37–8.95) |
|  |  | Eating disorder | 0.16  (0.16–0.17) | 73.54  (69.01–78.07) | 12.02  (7.44–16.61) | 0.16  (0.15–0.16) |  | 11.59  (6.95–16.23) |
|  |  | Dependence of alcohol | 0.15  (0.13–0.16) | 3.89  (3.08–4.70) | 0.58  (-0.22–1.39) | 0.15  (0.13–0.17) |  | 0.57  (-0.23–1.38) |
|  |  | Dependence of tobacco | 0.18  (0.17–0.19) | 10.61  (8.80–12.42) | 1.91  (0.10–3.71) | 0.18  (0.16–0.19) |  | 1.87  (0.06–3.68) |
|  |  | Dependence of cannabis | 0.15  (0.11–0.19) | 0.73  (0.39–1.06) | 0.11  (-0.22–0.45) | 0.17  (0.11–0.23) |  | 0.12  (-0.22–0.46) |
|  |  | Physical illnesses |  |  |  | 0.14  (0.13–0.14) | 115.70  (110.11–121.28) | 15.62  (9.81–21.43) |
| Boys | 13 | Anxiety | 0.16  (0.15–0.17) | 40.32  (35.30–45.33) | 6.36  (1.29–11.44) | 0.15  (0.14–0.17) |  | 6.23  (1.09–11.37) |
|  |  | Depression with suicidal ideation | 0.23  (0.21–0.24) | 15.67  (12.62–18.73) | 3.54  (0.48–6.60) | 0.21  (0.19–0.24) |  | 3.33  (0.25–6.40) |
|  |  | Depression without suicidal ideation | 0.19  (0.17–0.21) | 13.09  (10.48–15.70) | 2.44  (-0.18–5.06) | 0.19  (0.16–0.22) |  | 2.50  (-0.14–5.13) |
|  |  | Eating disorder | 0.13  (0.11–0.14) | 20.63  (17.06–24.19) | 2.67  (-0.90–6.25) | 0.14  (0.12–0.16) |  | 2.87  (-0.73–6.47) |
|  |  | Dependence of alcohol | 0.04  (-0.05–0.12) | 0.77  (0.15–1.38) | 0.03  (-0.58–0.64) | 0.00  (-0.06–0.07) |  | 0  (-0.61–0.61) |
|  |  | Dependence of tobacco | 0.12  (0.07–0.17) | 2.06  (0.90–3.22) | 0.26  (-0.90–1.41) | 0.14  (0.09–0.19) |  | 0.29  (-0.87–1.45) |
|  |  | Dependence of cannabis | 0.17  (0.10–0.24) | 0.99  (0.30–1.67) | 0.16  (-0.53–0.85) | 0.13  (0.10–0.16) |  | 0.13  (-0.56–0.82) |
|  |  | Physical illnesses |  |  |  | 0.10  (0.09–0.11) | 103.33  (93.49–113.18) | 10.10  (-0.15–20.35) |
|  | 14 | Anxiety | 0.16  (0.15–0.17) | 33.25  (29.51–36.98) | 5.45  (1.67–9.23) | 0.16  (0.15–0.17) |  | 5.26  (1.45–9.07) |
|  |  | Depression with suicidal ideation | 0.23  (0.21–0.25) | 15.45  (12.76–18.14) | 3.56  (0.85–6.26) | 0.24  (0.22–0.26) |  | 3.68  (0.97–6.39) |
|  |  | Depression without suicidal ideation | 0.20  (0.19–0.22) | 13.09  (10.65–15.52) | 2.67  (0.22–5.11) | 0.21  (0.19–0.23) |  | 2.73  (0.28–5.18) |
|  |  | Eating disorder | 0.12  (0.11–0.14) | 21.67  (18.52–24.83) | 2.71  (-0.46–5.87) | 0.13  (0.12–0.15) |  | 2.88  (-0.30–6.06) |
|  |  | Dependence of alcohol | 0.09  (0.06–0.12) | 3.12  (1.67–4.56) | 0.30  (-1.16–1.75) | 0.10  (0.07–0.13) |  | 0.31  (-1.14–1.76) |
|  |  | Dependence of tobacco | 0.12  (0.10–0.14) | 7.31  (4.54–10.08) | 0.90  (-1.87–3.67) | 0.11  (0.09–0.13) |  | 0.80  (-1.97–3.57) |
|  |  | Dependence of cannabis | 0.11  (0.06–0.16) | 1.48  (0.71–2.24) | 0.16  (-0.60–0.93) | 0.12  (0.07–0.16) |  | 0.17  (-0.59–0.94) |
|  |  | Physical illnesses |  |  |  | 0.10  (0.09–0.11) | 91.49  (84.25–98.72) | 9.29  (1.77–16.80) |
|  | 15 | Anxiety | 0.18  (0.17–0.19) | 26.67  (22.83–30.51) | 4.70  (0.84–8.56) | 0.16  (0.15–0.18) |  | 4.36  (0.47–8.24) |
|  |  | Depression with suicidal ideation | 0.24  (0.22–0.26) | 15.08  (12.11–18.05) | 3.56  (0.58–6.53) | 0.24  (0.21–0.27) |  | 3.63  (0.65–6.62) |
|  |  | Depression without suicidal ideation | 0.22  (0.20–0.24) | 10.49  (8.38–12.61) | 2.26  (0.13–4.39) | 0.22  (0.19–0.24) |  | 2.27  (0.14–4.41) |
|  |  | Eating disorder | 0.12  (0.11–0.14) | 17.62  (14.53–20.72) | 2.19  (-0.90–5.29) | 0.12  (0.11–0.14) |  | 2.20  (-0.91–5.31) |
|  |  | Dependence of alcohol | 0.10  (0.08–0.11) | 9.85  (7.23–12.46) | 0.95  (-1.66–3.57) | 0.09  (0.08–0.11) |  | 0.91  (-1.71–3.53) |
|  |  | Dependence of tobacco | 0.13  (0.12–0.14) | 17.21  (13.92–20.50) | 2.25  (-1.04–5.54) | 0.12  (0.11–0.14) |  | 2.11  (-1.20–5.41) |
|  |  | Dependence of cannabis | 0.10  (0.07–0.14) | 2.98  (1.84–4.12) | 0.31  (-0.83–1.44) | 0.13  (0.09–0.18) |  | 0.40  (-0.74–1.54) |
|  |  | Physical illnesses |  |  |  | 0.11  (0.10–0.11) | 79.59  (72.12–87.06) | 8.39  (0.67–16.10) |
| All boys | | Anxiety | 0.16  (0.16–0.17) | 33.26  (30.58–35.94) | 5.48  (2.78–8.17) | 0.16  (0.15–0.16) |  | 5.24  (2.53–7.95) |
|  |  | Depression with suicidal ideation | 0.23  (0.22–0.24) | 15.39  (13.54–17.24) | 3.56  (1.71–5.41) | 0.23  (0.22–0.24) |  | 3.53  (1.68–5.39) |
|  |  | Depression without suicidal ideation | 0.20  (0.19–0.21) | 12.20  (10.72–13.67) | 2.45  (0.98–3.93) | 0.20  (0.19–0.22) |  | 2.50  (1.02–3.98) |
|  |  | Eating disorder | 0.13  (0.12–0.13) | 19.94  (17.79–22.09) | 2.52  (0.37–4.67) | 0.13  (0.12–0.14) |  | 2.66  (0.50–4.81) |
|  |  | Dependence of alcohol | 0.09  (0.08–0.11) | 4.68  (3.56–5.80) | 0.44  (-0.68–1.56) | 0.09  (0.08–0.10) |  | 0.42  (-0.70–1.54) |
|  |  | Dependence of tobacco | 0.13  (0.12–0.14) | 9.03  (7.32–10.74) | 1.15  (-0.56–2.86) | 0.12  (0.11–0.13) |  | 1.08  (-0.63–2.79) |
|  |  | Dependence of cannabis | 0.11  (0.08–0.14) | 1.84  (1.31–2.36) | 0.21  (-0.32–0.74) | 0.13  (0.10–0.16) |  | 0.24  (-0.29–0.77) |
|  |  | Physical illnesses |  |  |  | 0.10  (0.10–0.11) | 91.20  (86.13–96.28) | 9.23  (4.00–14.45) |
| All | | Anxiety | 0.18  (0.17–0.18) | 72.52  (68.71–76.33) | 12.93  (9.08–16.78) | 0.17  (0.17–0.18) |  | 12.46  (8.58–16.34) |
|  |  | Depression with suicidal ideation | 0.24  (0.24–0.25) | 32.82  (30.48–35.16) | 8.04  (5.69–10.39) | 0.24  (0.24–0.25) |  | 7.98  (5.63–10.34) |
|  |  | Depression without suicidal ideation | 0.22  (0.21–0.23) | 20.31  (18.90–21.73) | 4.47  (3.05–5.89) | 0.22  (0.21–0.23) |  | 4.49  (3.06–5.92) |
|  |  | Eating disorders | 0.15  (0.15–0.16) | 46.58  (43.78–49.37) | 7.10  (4.29–9.91) | 0.15  (0.14–0.15) |  | 6.98  (4.15–9.80) |
|  |  | Dependence of alcohol | 0.12  (0.11–0.13) | 4.29  (3.52–5.05) | 0.50  (-0.26–1.27) | 0.11  (0.10–0.13) |  | 0.48  (-0.28–1.25) |
|  |  | Dependence of tobacco | 0.15  (0.15–0.16) | 9.81  (8.38–11.25) | 1.51  (0.07–2.94) | 0.15  (0.14–0.16) |  | 1.44  (0.01–2.88) |
|  |  | Dependence of cannabis | 0.12  (0.10–0.14) | 1.28  (0.94–1.63) | 0.15  (-0.20–0.51) | 0.13  (0.10–0.16) |  | 0.17  (-0.19–0.52) |
|  |  | Physical illnesses |  |  |  | 0.12  (0.11–0.12) | 103.37  (99.53–107.21) | 12.26  (8.28–16.23) |

**Note.** DW = Disability weights; Pyrs/1000 = person years per thousand population; YLD/1000 = years lived with disability per thousand population; CI = confidence interval.

^a^ For the calculation of years lived with disability in the subsample, the person years per thousand population of the total sample were used for each disorder, with the exception of physical illnesses, which was only completed in the subsample.
